# The first reported case of pseudoparathryoidism type IB in Hong Kong: hypocalcemia in an adolescent boy with recurrent seizures

**DOI:** 10.1186/1687-9856-2013-S1-P157

**Published:** 2013-10-03

**Authors:** Antony CC Fu, Kwok-lap Chan, Luke Chi-tak Tong

**Affiliations:** 1 Department of Paediatrics, The Chinese University of Hong Kong, Prince of Wales Hospital, Shatin, Hong Kong; 2 Department of Paediatrics, Alice Ho Miu Ling Nethersole Hospital

## 

Pseudohypoparathyroidism (PHP) is a heterogeneous group of disorders characterized by hypocalcemia, hyperphoshatemia and inappropriately elevated serum parathyroid hormone (PTH) level due to end-organ resistance of biological activity of parathyroid hormone. It is due to epigenetic change at the GNAS locus and is divided into types Ia, Ib, Ic and II. We hereby describe the first reported case of pseudohypoparathyroidism type Ib Hong Kong.

